# Temporal and spatial characterization of myopia in China

**DOI:** 10.3389/fpubh.2022.896926

**Published:** 2022-08-16

**Authors:** Xiujing Shi, Zhaorong Gao, Lin Leng, Zhen Guo

**Affiliations:** ^1^Qingdao Eye Hospital of Shandong First Medical University, Eye Institute of Shandong First Medical University, Qingdao, China; ^2^State Key Laboratory Cultivation Base, Shandong Provincial Key Laboratory of Ophthalmology, Eye Institute of Shandong First Medical University, Qingdao Eye Hospital of Shandong First Medical University, Qingdao, China; ^3^School of Ophthalmology, Shandong First Medical University, Qingdao, China

**Keywords:** myopia, geographic information system, spatial autocorrelation analysis, spatiotemporal analysis, China

## Abstract

**Purpose:**

The aim of this study was to characterize the temporal and spatial distribution of myopia among students aged 7–18 years, by analyzing the aggregation area and providing the basis for the prevention and control of myopia in China.

**Methods:**

A database for the spatial analysis of myopia in China during 1995–2014 was established using ArcGIS10.0 software as a platform for data management and presentation. A spatial autocorrelation analysis of myopia was undertaken, and a temporal and spatial scan analysis was performed using SaTScan9.5 software.

**Results:**

Our data demonstrated that the prevalence of myopia in China in 1995, 2000, 2005, 2010, and 2014 was 35.9, 41.5, 48.7, 57.3, and 57.1%, respectively, thus indicating a gradual upward trend. The prevalence of myopia was analyzed in various provinces (municipalities and autonomous regions), and the highest was found in Jiangsu Province, with an average Moran's *I* index of 0.244295 in China (*P* ≤ 0.05). According to the local Moran's *I* autocorrelation analysis, there was a spatial aggregation of myopia prevalence among students in the entire country, with Shandong, Jiangsu, Anhui, and Shanghai being classified as high–high aggregation areas, while Hainan and Guangxi were classified as low–low aggregation areas. In addition, the Getis-Ord General *G* results of the global hotspot analysis showed a countrywide myopia prevalence index of 0.035020 and a *Z* score of 1.7959 (*P* = 0.07251). Because the myopia prevalence correlation difference was not statistically significant, there were no “positive hotspots” or “negative hotspots.” The local hotspot analysis shows that Shandong and Jiangsu belong to high-value aggregation areas, while Hainan and Guizhou belong to low-value aggregation areas. Further analysis using time-space scanning showed 15 aggregation regions in five stages, with four aggregation regions having statistically significant differences (*P* ≤ 0.05). However, the aggregation range has changed over time. Overall, from 1995 to 2014, the aggregation areas for the myopia prevalence in Chinese students have shifted from the northwest, north, and northeast regions to the southeast regions.

**Conclusion:**

Our data demonstrate that, from 1995 to 2014, the prevalence of myopia increased in students aged 7–18 years in China. In addition, the prevalence of myopia is randomly distributed in various provinces (municipalities and autonomous regions) and exhibits spatial aggregation. Also, the gathering area is gradually shifting to the southeast, with the existence of high-risk areas. It is, therefore, necessary to focus on this area and undertake targeted prevention and control measures.

## Introduction

Myopia, a worldwide eye disease, affects 28% of the world's population and is associated with visual impairment (1). China is one of the countries with the highest incidence of myopia (2, 3). At present, there is a high prevalence of myopia among adolescents in China, as well as a significant disease progression. According to statistics, the prevalence of myopia among Chinese children and adolescents nationwide was 52.7% in 2020, and China's Ministry of Education issued an online survey to 14,532 students in nine provinces, which indicated that myopia prevalence increased by 11.7% in 2020 as compared with the end of 2019 (4, 5). Myopia not only endangers the eyesight of teenagers but also affects the mental health, lifestyle, and quality of life of families and society (6, 7). Myopia has become a global public health concern, and the World Health Organization (WHO) has incorporated myopia prevention and control measures into its global blindness prevention plan (8). In addition, the WHO has listed myopia as one of the five types of eye diseases that aims to improve and eliminate (9). It is, therefore, imperative to enhance educational reforms and develop effective and comprehensive prevention and control programs, as well as encourage the whole society to take action against visual impairment in children.

One important measure to enable the effective prevention of myopia is understanding the modifiable risk factors from a public perspective (10). Numerous studies have shown that many factors seem to contribute to the pathogenesis of myopia. However, considering the high prevalence of myopia, there is an increasing need to explore other environmental factors that may affect its prevalence. A previous study (11) showed that the incidence of myopia is associated with geography, population dynamics, and the economy. It is, therefore, important to characterize the spatial distribution and epidemic trend regarding myopia so as to inform the development of effective prevention and control measures (1–3).

A geographic information system (GIS) is a technical system based on a geospatial database and is supported by computer hardware and software systems that can collect, store, manage, compute, analyze, display, and describe the relevant geographical distribution of data in space (12). The use of GIS enables the convenient description and analysis of the spatial and temporal distribution patterns of population diseases, health, and health events (13). In addition, GIS enables the exploration of factors affecting the health status of specific populations, which helps in disease prevention and control, health promotion, and health services (14, 15).

In this study, we used GIS to analyze the spatial distribution and gather situations regarding myopia prevalence among students aged 7–18 years at the provincial level in China from 1995 to 2014. Our study offers critical insights and provides the basis for further research on myopia prevention and control strategies.

## Materials and methods

### Sources of materials

The prevalence of myopia from 1995 to 2014 was retrieved from the Report on Chinese Students' Physique and Health (1995, 2000, 2005, 2010, and 2014). The research object was students aged 7–18 years from various provinces and cities in mainland China. The stratified random cluster sampling method was used to select the survey samples. First, the province was divided into three areas (i.e., good, medium, and poor) according to the gross domestic product and other factors; second, the students were divided into 12 groups according to their age (7–18 years); finally, ≥50 students were randomly sampled in each area and from each age group. According to four categories (i.e., urban, rural, male, and female), the sample size for each province should be ≥50^*^4^*^12^*^3 = 7,200 students. After excluding students with abnormal or missing values, the final test sample in each province is about 7,000 students. The detailed sample size for each province has been described in the Report on Chinese Students' Physique and Health and presented in Table 1 (16–20). The five surveys were all inspected by professionally trained inspectors and completed between September and October of the year in question. In accordance with the unified requirements of the National Student Physique and Health Survey Work Manual, the on-site quality control was carried out by supervisors.

**Table 1:** The number of participants in each province in the survey from 1995 to 2014.

**Ranking**	**Province (district, municipality)**	**1995**	**2000**	**2005**	**2010**	**2014**
1	Beijing	7,473	7,297	7,577	7,198	7,000
2	Tianjin	7,201	4,320	7,490	7,198	7,183
3	Hebei	7,200	6,621	7,904	6,921	7,187
4	Shanxi	7,210	7,189	7,091	7,192	7,200
5	Inner Mongolia	5,760	6,906	7,707	7,180	7,058
6	Liaoning	8,156	12,581	7,218	7,179	7,190
7	Jilin	7,209	7,342	8,587	7,168	7,114
8	Heilongjiang	7,683	7,188	7,124	7,173	7,175
9	Shanghai	7,919	7,362	6,404	7,200	7,143
10	Jiangsu	7,676	7,529	9,237	6,335	6,949
11	Zhejiang	8,416	7,195	7,211	7,192	6,825
12	Anhui	7,200	7,199	7,184	7,198	7,196
13	Fujian	5,733	7,121	7,690	7,179	7,200
14	Jiangxi	7,172	7,113	7,441	7,142	7,174
15	Shandong	7,198	8,487	8,570	7,135	7,178
16	Henan	8,639	7,192	8,620	7,200	7,200
17	Hubei	7,198	7,194	4,684	7,098	7,067
18	Hunan	8,314	7,191	7,381	7,165	7,196
19	Guangdong	7,200	7,194	7,194	7,199	7,189
20	Guangxi	8,662	7,204	7,189	7,088	6,905
21	Hainan	7,134	6,548	9,681	7,079	7,200
22	Sichuan	8,640	8,136	8,978	7,138	7,198
23	Guizhou	7,200	7,192	7,179	7,182	7,197
24	Yunnan	6,673	4,991	7,841	7,195	7,200
25	Chongqing	/	7,183	9,199	7,183	7,200
26	Shanxi	7,679	7,199	7,193	7,189	7,181
27	Gansu	8,545	8,025	8,472	7,199	7,196
28	Qinghai	/	2,401	7,379	7,165	7,197
29	Ningxia	7,199	7,200	7,430	6,975	6,879
30	Xinjiang	7,198	2,400	10,253	7,189	5,966
The whole nation		**209,487**	**208,700**	**235,505**	**216,474**	**215,160**

The 5M Standard Logarithmic Visual Chart was used to test the students' uncorrected visual acuity (21). A vision score of 5.0 is normal, and 4.9 indicates poor vision. The vision of subjects with a visual acuity below 4.9 was corrected with lenses so as to reach 5.0 visual acuity. If the applied lenses had a negative lens power, those students were chosen as myopic, and the accommodative accuracy was further ensured using retinoscopy. Students with ≤ -0.5D were included in the myopia group in this study.

The Report on Chinese Students' Physique and Health was compiled by the Chinese Students' Physique and Health Research Group and has been reviewed by the Ministry of Education, the National Health Commission of the People's Republic of China, and other departments. The report was open to the public.

The national myopia data exploited the national provincial vector map (1:1.6 million) and established serial numbers for each province (municipality and autonomous region) from the attribute database. The sorted prevalence of myopia was associated and matched with the serial number on the basic map so as to establish a complete spatial analysis database.

### Methods

#### Spatial autocorrelation analysis

The global and local Moran's *I* indexes were used to explore the spatial autocorrelation of the prevalence of myopia. The spatial weight matrix was generated using the spatial conceptualization method based on inverse distance. We then used ArcGIS 10.2 software to calculate the global and local Moran's *I* indexes on the basis of the defined weight matrix. In the Moran's *I* matrix, the larger the *I*-value, the greater the correlation of spatial distribution and, thus, the more obvious the phenomenon of spatial aggregation distribution. On the contrary, as the *I*-value approaches 0, the spatial distribution is random (22). In addition, we used a test statistic *Z*-value of approximately normal distribution under random conditions. A *P*-value of < 0.05 was taken to indicate statistical significance. We used the global spatial autocorrelation to analyze whether the study indicated aggregated distribution in general and the local spatial autocorrelation to describe the correlation between the prevalence of myopia in each province (municipality and autonomous region) and its neighboring provinces (municipality and autonomous region). We then defined the specific aggregation areas and aggregation mode (23).

#### Hotspot analysis (Getis-Ord G)

We used the global *G* statistics to evaluate the presence of “positive hotspots” or “negative hotspots” in the prevalence of myopia. Briefly, a *G* of more than 0 implies high-value aggregation regions, and *vice versa*, with a *P* of <0.05 in the research range (24). On the contrary, we used the local *G* statistics to evaluate the high- or low-value aggregation areas in all provinces (municipalities and autonomous regions) and neighboring provinces (municipalities and autonomous regions). A *Z*-value of more than 1.96 suggested a high-value aggregation area in and around the spatial unit i, while a *Z*-value of < -1.96 suggested the opposite (25).

#### Spatio-temporal scanning analysis

To ensure an accurate description of the distribution of temporal and spatial aggregation of the prevalence of myopia, we divided five surveys of the Chinese student system into five stages based on the spatial autocorrelation analysis (26). SaTScan9.5 software was used for the aggregation analysis, while ArcGIS10.2 software was used for the visualization of the results (27). The data on the prevalence of myopia in 31 provinces (municipalities and autonomous regions) from 1995 to 2014 were analyzed by visualizing the spatial distribution in ArcMap. The SaTScan 9.6 software detected the spatial aggregation of the disease in the research area through a series of scanning circles. Because the size and position of the window were dynamic, the statistical inference was made by calculating the log likelihood ratio (LLR) values of different spatial unit attributes within and outside the dynamic window regions. A large LLR value implied that the area under the window was likely to have aggregation (28). The spatial-temporal scanning analysis of this study adopted the space-time permutation model in the SaTScan 9.5 software. We adopted high-value clustering, the number of Monte Carlo simulation tests at 999, and the clustering of time interval at 5 years.

## Results

### Spatial distribution of the prevalence of myopia

**Figure 1:**
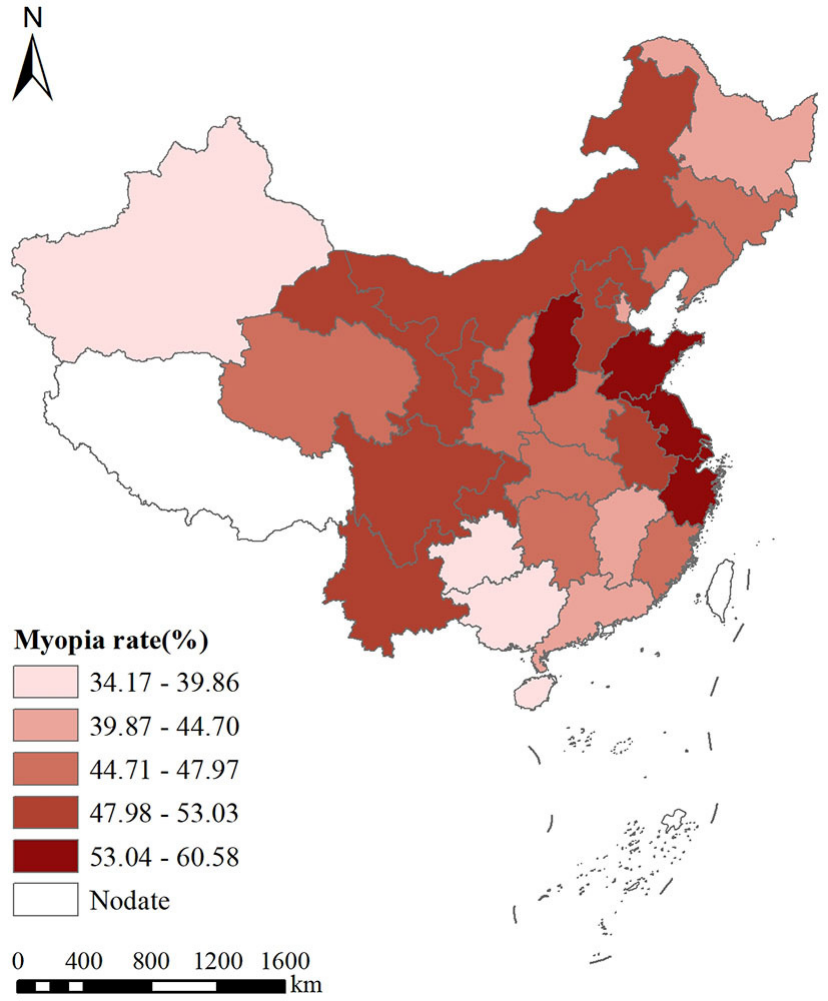
Spatial distribution of the prevalence of myopia in China from 1995 to 2014.

The prevalence of myopia was 35.9, 41.5, 48.7, 57.3, and 57.1% in 1995, 2000, 2005, 2010, and 2014, respectively. Jiangsu and Hainan Provinces had the highest and lowest prevalence of myopia, respectively, among the provinces (municipalities and autonomous regions). The average prevalence of myopia from 1995 to 2014 was divided into five grades according to the natural breakpoint classification method. Hainan, Guizhou, Xinjiang, and Guangxi had the lowest myopia prevalence and are assigned to the first grade, while Jiangsu, Shandong, Shanghai, Zhejiang, and Shanxi had the highest myopia prevalence, thus ranking fifth, as shown in Figure 1 and Table 2.

**Table 2:** The prevalence of myopia among children and adolescents in all provinces of China from 1995 to 2014.

**Ranking**	**Province (district, municipality)**	**1995**	**2000**	**2005**	**2010**	**2014**
1	Beijing	33.77%	40.63%	51.78%	60.20%	66.40%
2	Tianjin	24.42%	32.64%	43.68%	42.33%	66.30%
3	Hebei	41.72%	47.82%	51.71%	62.70%	41.70%
4	Shanxi	49.29%	46.46%	58.07%	68.48%	57.60%
5	Inner Mongolia	33.26%	35.35%	48.59%	68.38%	64.20%
6	Liaoning	33.46%	35.91%	54.62%	58.64%	56.90%
7	Jilin	34.24%	37.44%	46.40%	56.31%	58.20%
8	Heilongjiang	23.82%	31.00%	46.91%	54.36%	55.00%
9	Shanghai	45.84%	49.38%	59.96%	69.00%	71.80%
10	Jiangsu	43.55%	51.56%	66.02%	69.75%	72.00%
11	Zhejiang	52.57%	56.00%	63.09%	72.97%	47.90%
12	Anhui	34.93%	48.84%	45.80%	57.47%	63.00%
13	Fujian	41.82%	47.40%	31.04%	60.57%	57.10%
14	Jiangxi	34.80%	40.11%	41.03%	51.91%	52.20%
15	Shandong	46.75%	50.74%	63.56%	69.43%	66.90%
16	Henan	35.15%	39.77%	43.73%	55.85%	57.90%
17	Hubei	37.81%	41.52%	45.64%	55.13%	52.00%
18	Hunan	33.22%	39.05%	50.11%	52.85%	57.80%
19	Guangdong	29.92%	34.62%	45.28%	55.10%	58.60%
20	Guangxi	28.15%	33.56%	43.72%	51.65%	42.20%
21	Hainan	26.15%	27.92%	36.18%	39.38%	41.25%
22	Sichuan	39.14%	48.21%	57.33%	57.28%	63.20%
23	Guizhou	25.53%	29.10%	32.58%	50.30%	45.90%
24	Yunnan	36.72%	44.76%	49.78%	55.75%	64.30%
25	Chongqing	None	39.02%	52.29%	53.38%	56.00%
26	Shanxi	33.60%	41.48%	50.33%	56.02%	58.40%
27	Gansu	38.87%	42.90%	57.06%	57.28%	65.60%
28	Qinghai	None	42.61%	38.65%	54.97%	48.20%
29	Ningxia	39.76%	37.69%	55.16%	54.77%	61.60%
30	Xinjiang	26.50%	43.79%	39.37%	47.90%	39.80%
The whole nation		**35.93%**	**41.54%**	**48.71%**	**57.35%**	**57.09%**

### Spatial autocorrelation analysis

#### Global spatial autocorrelation analysis

Our data demonstrated that the Moran's *I* indexes for the students' prevalence of myopia were 0.101352, 0.255234, 0.140538, 0.169005, and 0.087811 in 1995, 2000, 2005, 2010, and 2014, respectively, with all indexes having a *P* < 0.05. The average Moran's *I* index was 0.244295. Because the difference in autocorrelation within the regional range was statistically significant, there was a positive spatial correlation regarding the prevalence of myopia in China (Table 3).

**Table 3:** Global spatial autocorrelation analysis results for myopia prevalence in China from 1995 to 2014.

**Year**	**Moran's *I* index**	***Z*** **score**	***P*** **-value**
1995	0.101352	1.685217	0.091947
2000	0.255234	3.375541	0.000737
2005	0.140538	2.044891	0.040866
2010	0.169005	2.401253	0.016339
2014	0.087811	1.424233	0.154379
Average	0.244295	3.276747	0.001050

#### Local spatial autocorrelation analysis

**Figure 2:**
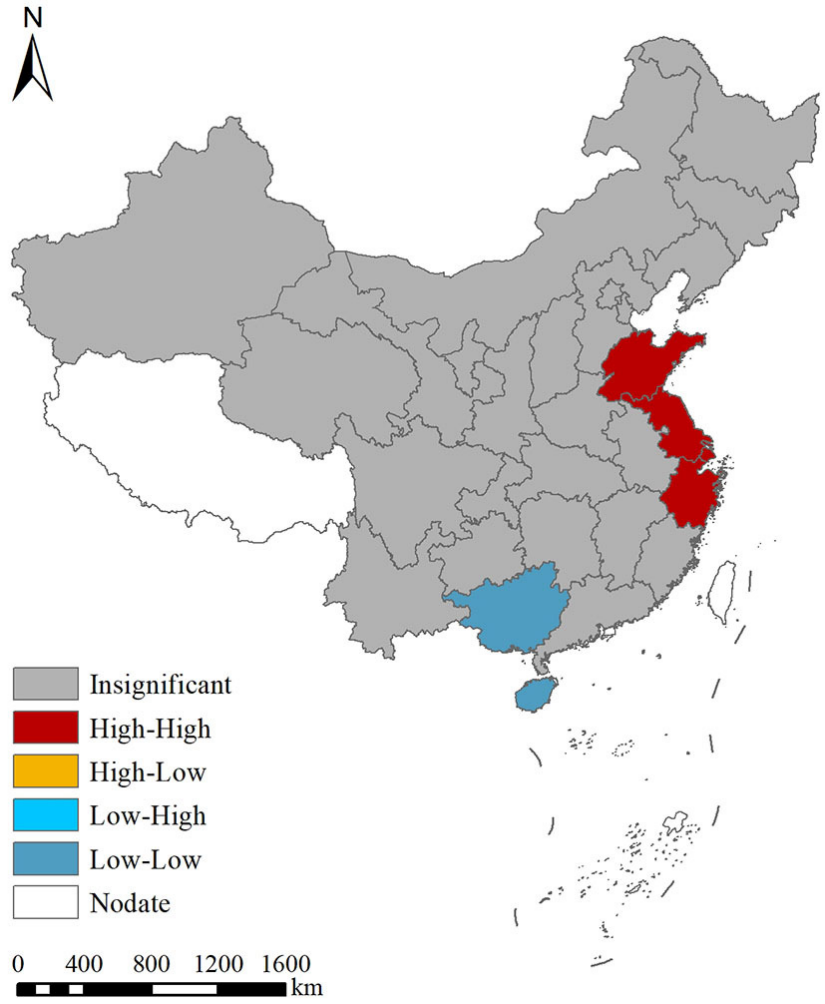
Local spatial autocorrelation analysis for the prevalence of myopia in China from 1995 to 2014.

The local Moran's *I* autocorrelation analysis shows that there was a spatial aggregation regarding the prevalence of myopia among students nationwide (Figure 2). Shandong, Jiangsu, Anhui, and Shanghai were classified as high–high aggregation areas, while Hainan and Guangxi were classified as low–low aggregation areas.

#### Global and local hotspot analysis

**Figure 3:**
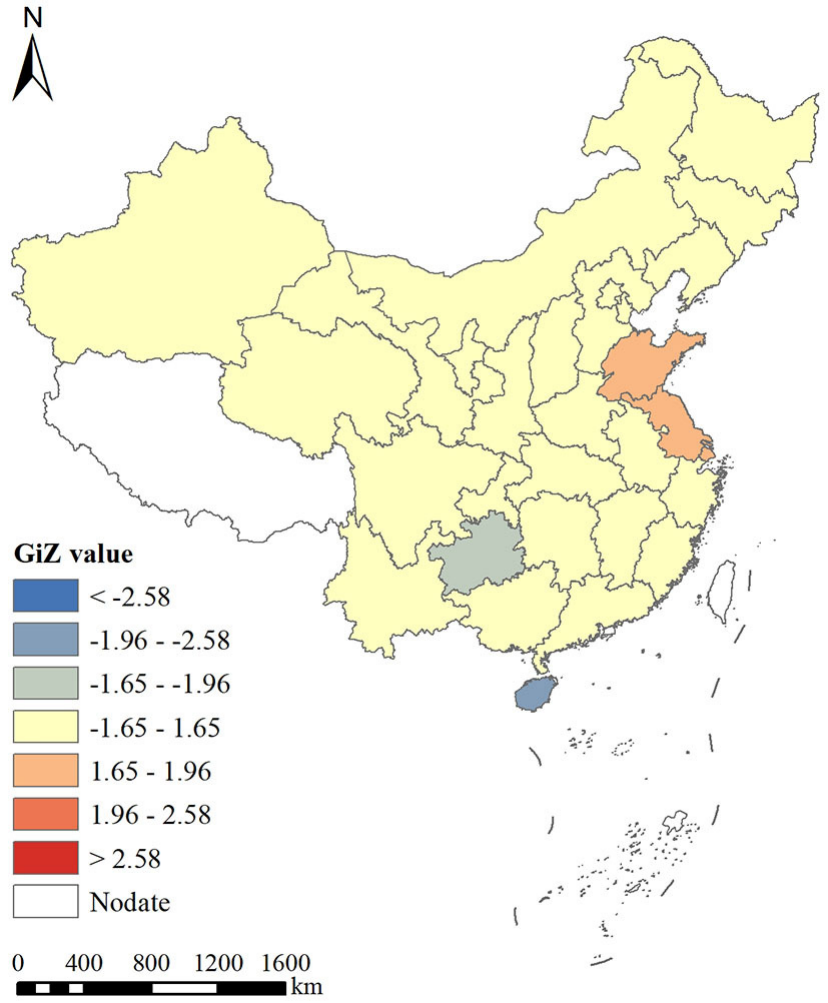
Hotspot analysis of average annual myopia prevalence in China from 1995 to 2014.

Our Getis-Ord General *G* results for the global hotspot analysis showed that the general *G* index for the prevalence of myopia was 0.035020, while the *Z* and *P*-values were 1.795897 and 0.072511, respectively. These data demonstrated a lack of statistical significance in the correlation of the prevalence of myopia within the region, as well as a lack of either “positive” or “negative” hot spots. In addition, the local hotspot analysis showed that Shandong and Jiangsu belong to a high-value aggregation area, while Hainan and Guizhou belong to a low-value aggregation area (Figure 3).

### Spatio-temporal scanning analysis

**Figure 4:**
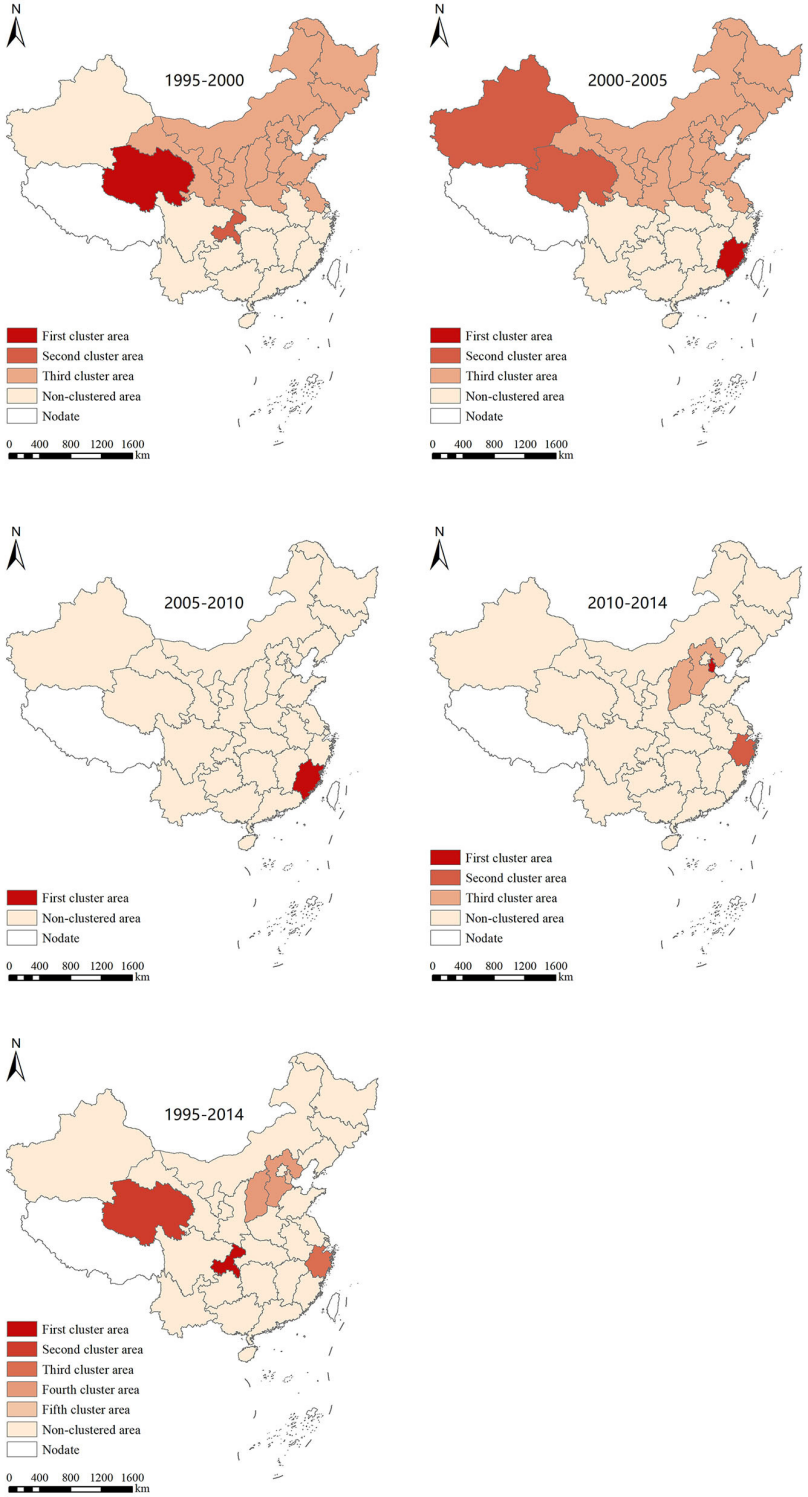
Spatial and temporal scanning aggregation area for the prevalence of myopia in Chinese students from 1995 to 2014.

Following the temporal and spatial scanning analysis of myopia prevalence, we realized a total of 15 aggregation areas in five stages, with obvious changes in the aggregation areas. From 1995 to 2000, the first-level gathering area was largest in 2000 and involved only Qinghai, while the second-level aggregation area was in Chongqing. The third aggregation area was mainly distributed in North and Northeast China. From 2000 to 2005, Fujian or Xinjiang and Qinghai became the first and the second aggregation areas, respectively. From 2005 to 2010, the aggregation area was mainly concentrated in Fujian. From 2010 to 2014, the first and the second aggregation areas were Tianjin and Zhejiang, while the third aggregation area included Shanxi and Hebei. On the contrary, from 1995 to 2014, the aggregation areas saw an overall shift from the northern regions to the southeast regions (Figure 4 and Table 4).

**Table 4:** Temporal and spatial scanning aggregation analysis of myopia prevalence in China from 1995 to 2014.

**Time phase**	**Aggregation area**	**Province and municipality**	**Aggregation time**	**RR value**	**LLR value**	***P*** **-value**
1995–2000	1	Qinghai	2000	1.81	6.38	0.000
	2	Chongqing	2000	1.81	5.78	0.000
	3	Heilongjiang, Inner Mongolia, Jilin, Gansu, Beijing, Shanxi, Shanxi, Ningxia, Henan, Hebei, Liaoning, Shandong, Tianjin, Jiangsu	1995	1.05	0.88	0.934
2000–2005	1	Fujian	2000	1.32	1.66	0.382
	2	Xinjiang, Qinghai	2000	1.15	0.87	0.916
	3	Heilongjiang, Inner Mongolia, Jilin, Gansu	2005	1.04	0.71	0.977
2005–2010	1	Fujian	2010	1.23	1.25	0.794
2010–2014	1	Tianjin	2014	1.23	1.29	0.725
	2	Zhejiang	2010	1.20	1.20	0.783
	3	Shanxi, Hebei	2010	1.13	1.00	0.913
1995–2014	1	Chongqing	2014	1.59	5.26	0.001
	2	Qinghai	2014	1.59	4.49	0.004
	3	Zhejiang	2010	1.42	2.91	0.059
	4	Shanxi, Hebei	2010	1.29	2.72	0.080
	5	Tianjin	2014	1.17	0.75	0.980

## Discussion

The eye health of adolescents is an important aspect of national health. Adolescent myopia not only creates inconvenience in terms of one's personal life and studies but also increases the burden on society and one's family. Therefore, myopia is a major public health and social issue affecting people's livelihoods (29–34). The age between 7 and 18 years is critical for eye development; thus, it represents an important phase in which to protect and prevent the development of poor eyesight. Whereas myopia is a serious public health problem all over the world, the geographical vastness of China increases the complexity of myopic students in China (35–38).

An accurate characterization of the spatial distribution defines the myopia distribution dynamics and influencing factors and thus might inform effective prevention and control measures (27, 28). In this study, we used the spatial analysis technology of GIS to interrogate the spatial and temporal distribution of the prevalence of myopia among students in various provinces, as well as explore the hotspot regions. In the process of myopia prevention and control in our country, time, space, and social factors are all crucial. Therefore, the distribution of the students' prevalence of myopia shows a certain degree of spatial heterogeneity in different areas (39–41). This study analyzed the temporal and spatial characteristics of the prevalence of myopia among students in China from 1995 to 2014 at the provincial level. The spatial distribution analysis showed that the prevalence of myopia among Chinese students was gradually increasing. The average prevalence of myopia from 1995 to 2014 was divided into five grades. Hainan, Guizhou, Xinjiang, and Guangxi had the lowest myopia prevalence, while Jiangsu, Shandong, Shanghai, Zhejiang, and Shanxi had the highest myopia prevalence. One potential explanation is that, given that Shandong and Jiangsu are located in the economically developed areas in eastern China, the rapid economic development and continuous improvement of education levels in the eastern coastal areas have led to high academic pressure on students, less time outdoors, and longer close-work hours (42, 43). A study (44) conducted in Anyang, China, by Wei et al. found that more time outdoors, close-work time, and time spent sleeping were associated with myopia in children.

However, our study used data only on visual acuity, and non-cycloplegic refractive errors have been shown to be problematic in epidemiological studies of myopia (45). Therefore, the results of this study may be biased. In addition, visual acuity was not adjusted for confounding factors, and we should improve on this aspect of the work in the next step.

From 1995 to 2014, there was no spatial positive correlation for the prevalence of myopia in students aged between 7 and 18 years. The local Moran's *I* autocorrelation analysis showed that there was a spatial aggregation of the students' myopia prevalence, with Shandong, Jiangsu, Anhui, and Shanghai being high–high aggregation areas and Hainan and Guangxi being low–low aggregation areas. On the contrary, the global hotspot analysis showed that there was no correlation difference regarding the students' myopia prevalence, with no “positive” or “negative” hotspot regions. The local hotspot analysis showed that Shandong and Jiangsu belonged to the high-value aggregation area, while Hainan and Guizhou belonged to the low-value aggregation area. This was related to the pressure of entering high school, heavy academic burden, and long reading times in Shandong and Jiangsu, while Hainan's low-value aggregation was related to its good living environment, broad vision on the part of students, backward local culture and economy, limited television watching time, small schoolwork burden, and sufficient extracurricular activities (46–48).

Because the spatial autocorrelation analysis could not determine the size and scope of aggregation, we employed the spatial-temporal scanning analysis. A total of 15 aggregation areas were found in the five stages. However, there was a shift in the aggregation area from the northwest, north, and northeast to the southeast. The unpublished data from our research group showed that the prevalence of myopia in children and adolescents in the eastern coastal areas showed a double-high trend, that is, a high prevalence of myopia and a high prevalence of high myopia. One potential explanation is that, as compared with the northwest region, the economic development speed of the southeast coastal area is fast, and the educational level is constantly improving, which leads to increased academic pressure on students. The gap between the southeast coastal region and the northwest region is increasing year by year, which leads to the gathering area gradually shifting to the southeast. In addition, as compared with the northwest region, the southeast coastal areas, with their developed economy and suitable environment, may attract more highly educated talents, and the prevalence of myopia in the next generation may be higher, which will also lead to a shift in the gathering area to the southeast coast (49, 50). It is suggested that we should focus on the occurrence and development of myopia in children and adolescents in the southeast coast and undertake timely intervention measures to protect children's and adolescents' eye health.

The occurrence and development of myopia are diverse and complex, with changes across time and space, as well as across age groups and learning stages. At present, the research on the prevention and control of myopia mainly focuses on two aspects. One aspect is the basic and clinical research; specifically, the effectiveness of atropine in the prevention and control of myopia has been preliminarily recognized (51). In addition, the orthokeratology lens has also been proven to be a safe, effective, and reversible intervention measure to halt myopia progress (52). Another aspect is myopia Big data research. The mechanism behind myopia remains unclear, and it is very important to clarify the process of children's refractive development and thus prevent and control myopia. The hyperopic reserve is considered an important indicator of the occurrence and development of myopia (53). However, the lack of data on the hyperopic reserve is a limitation of this study, and we will explore this issue in the next step.

Although there are many studies on the prevention and control of myopia, the current prevention effect is not significant. The most important issue for public health policies is the decrease in the academic load that has been established with the limitation of tutorial classes and a potential increase in time spent outdoors, which is very limited in those environments studied in this work (54). Therefore, it is urgent to build a diversified and interconnected myopia prevention and control network and thus create a mass myopia prevention and control mechanism for student-family-school-medical institutions. The whole society should work together to maintain the eye health of its children and adolescents.

## Limitations

However, this study also has several limitations. First, our data come from a research report on Chinese students' physique and health, but due to national policies, the report was only updated until 2014, and the eye health data for primary and secondary school students from 2015 to 2019 could not be obtained, which means our research results include a certain amount of lag. Second, although we identified established physical fitness screening systems for primary and secondary school students in China, most provinces did not publish their data collection procedures. As a result of this, the accuracy of the data obtained from these cannot be verified. Finally, there may be other factors that influenced the findings that were not taken into account.

## Conclusion

In conclusion, from 1995 to 2014, the prevalence of myopia in China shows an increasing trend over the years. The average annual myopia prevalence of each province (autonomous regions and municipality) is randomly distributed and has a certain spatial aggregation. The aggregation areas, based on phased spatio-temporal scanning, are increasing gradually and shifting from the northwest, north, and northeast to the southeast, where high-risk areas regarding myopia continue to exist. It is, therefore, necessary to focus on these areas and undertake targeted prevention and control measures.

## Data availability statement

The original contributions presented in the study are included in the article/supplementary material, further inquiries can be directed to the corresponding author.

## Ethics statement

Ethical review and approval was not required for the study on human participants in accordance with the local legislation and institutional requirements. Written informed consent to participate in this study was provided by the participants' legal guardian/next of kin. Written informed consent was obtained from the minor(s)' legal guardian/next of kin for the publication of any potentially identifiable images or data included in this article.

## Author contributions

XS and ZGa carried out the procedure including the literature search, data extraction, statistical analysis, and manuscript writing. LL conceived the study and revised the manuscript. ZGu participated in the method development and revised the manuscript. All authors read and approved the final manuscript.

## Funding

This work has been supported by the Chinese Academy of Engineering Consulting Research Project (2019-XY-84) and Shandong First Medical University (Shandong Academy of Medical Sciences) Academic Enhancement Program (2019ZL001).

## Conflict of interest

The authors declare that the research was conducted in the absence of any commercial or financial relationships that could be construed as a potential conflict of interest.

## Publisher's note

All claims expressed in this article are solely those of the authors and do not necessarily represent those of their affiliated organizations, or those of the publisher, the editors and the reviewers. Any product that may be evaluated in this article, or claim that may be made by its manufacturer, is not guaranteed or endorsed by the publisher.
